# Tiny microbes, enormous impacts: what matters in gut microbiome studies?

**DOI:** 10.1186/s13059-016-1086-x

**Published:** 2016-10-19

**Authors:** Justine Debelius, Se Jin Song, Yoshiki Vazquez-Baeza, Zhenjiang Zech Xu, Antonio Gonzalez, Rob Knight

**Affiliations:** 1 Department of Pediatrics, University of California San Diego, La Jolla, CA USA; 2 Department of Ecology and Evolutionary Biology, University of Colorado Boulder, Boulder, CO USA; 3 Department of Computer Science and Engineering, University of California San Diego, La Jolla, CA USA

## Abstract

Many factors affect the microbiomes of humans, mice, and other mammals, but substantial challenges remain in determining which of these factors are of practical importance. Considering the relative effect sizes of both biological and technical covariates can help improve study design and the quality of biological conclusions. Care must be taken to avoid technical bias that can lead to incorrect biological conclusions. The presentation of quantitative effect sizes in addition to *P* values will improve our ability to perform meta-analysis and to evaluate potentially relevant biological effects. A better consideration of effect size and statistical power will lead to more robust biological conclusions in microbiome studies.

## Introduction

The human microbiome is a virtual organ that contains >100 times as many genes as the human genome [1]. In the past 10 years, our understanding of associations between the microbiome and health has expanded greatly. Our microbial symbionts have been implicated in a broad range of conditions including: obesity [2, 3]; asthma, allergies, and autoimmune conditions [4–10]; depression (reviewed in [11, 12]) and other mental illnesses [13, 14]; neurodegeneration [15–17]; and vascular disease [18, 19]. Nevertheless, integrating this rapidly expanding literature to find general patterns is challenging because of the myriad ways in which differences are reported. For example, the term 'dysbiosis’ may reflect differences in alpha diversity (the biological diversity within a sample) [13], in beta diversity (the difference in microbial community structure between samples) [20], in the abundances of specific bacterial taxa [7, 14, 15], or any combination of these three components [4, 6]. All of these differences might reflect real kinds of dysbiosis, but studies that focus on different features are difficult to compare. Even drawing generalities from different analyses of alpha diversity can be complicated. It is well known that errors in sequencing and DNA sequence alignments can lead to substantial inflation of counts of the species apparent in a given sample [21–25]. Moreover, different measures of diversity focusing on richness (the number of kinds of entities), evenness (whether all entities in the sample have the same abundance distribution), or a combination of these can produce entirely different results than ranking samples by diversity.

Establishing consistent relationships between specific taxa and disease has been especially problematic, in part because of differences in how studies define clinical populations, handle sample preparation and DNA-sequencing methodology, and use bioinformatics tools and reference databases, all of which can affect the result substantially [26–29]. A literature search may find that the same taxon has been both positively and negatively associated with a disease state in different studies. For example, the Firmicutes to Bacteriodetes ratio was initially thought to be associated with obesity [30] and was considered a potential biomarker [31], but our recent meta-analysis showed no clear trend for this ratio across different human obesity studies [32]. Some of the problems could be technical, because differences in sample handling can change the observed ratio of these phyla [33] (although we would expect these changes to cause more issues when comparing samples between studies than when comparing those within a single study). Consequently, identifying specific microbial biomarkers that are robust across populations for obesity (although, interestingly, not for inflammatory bowel disease) remains challenging. Different diseases will likely require different approaches.

Despite problems in generalizing some findings across microbiome studies, we are beginning to understand how the effect size can help to explain differences in community profiling. In statistics, effect size is defined as a quantitative measure of the differences between two or more groups, such as a correlation coefficient between two variables or a mean difference in abundance between two groups. For example, the differences in overall microbiome composition between infants and adults are so large that they can be seen even across studies that use radically different methods [34]; this is because the relative effect size of age is larger than that of processing technique. Therefore, despite problems in generalizing findings across some microbiome studies that result from the factors noted above, we are beginning to understand how the effect sizes of specific biological and technical variables in community profiling are structured relative to others.

In this review, we argue that by explicitly considering and quantifying effect sizes in microbiome studies, we can better design experiments that limit confounding factors. This principle is well established in other fields, such as ecology [35], epidemiology (see for example [36]), and genome-wide association studies (their relationship to microbiome studies is reviewed in [37]). Avoiding important confounding variables that have a large effect size will allow researchers to more accurately and consistently draw meaningful biological conclusions from these studies of complex systems.

## Biological factors that affect the microbiome

Specific consideration of effect sizes is crucial for interpreting naturally occurring biological variation in the microbiome, where the effect being investigated is frequently confounded by other factors that might affect the observed community structure. Study designs must consider the relative scale of different biological effects (for example, microbiome changes induced by diet, drugs, or disease) and technical effects (for example, the effects of PCR primers or DNA extraction methods) when selecting appropriate controls and an appropriate sample size. To date, biological factors with effects on the microbiome of varying sizes have been observed (Table 1). Consider, for example, the effect of diet on the microbiome.

**Table 1:** The relative effects of biological covariates affecting the microbiome

Covariate	References	Findings
Large		
Host species	[41–44]	The gut microbiome of host species separates by dietary patterns and phylogeny. Animals that have diets that diverge from those of their ancestors have microbiomes that are adapted to their new diets.
Age	[45, 46, 52]	Infants have dramatically different microbiomes to adults, and undergo a rapid period of developmental maturation. After the introduction of solid food, the microbiomes of older children begin to resemble those of their parents and move toward an adult community structure.
Lifestyle	[45, 54]	Western adults and adults living traditional lifestyles (e.g., agriculturists, hunter-gatherers) have large differences in their microbiomes.
Medium		
Antibiotic use	[55, 56, 58, 59]	Antibiotics have a sustained effect on the microbiome, leading to altered community structure and lower alpha diversity. Individualized responses to the same antibiotic vary, and different antibiotics may have different impacts.
Medium to small; difficult to rank		
Long-term dietary patterns	[61, 62]	A low-fiber diet leads to the loss of species, although diversity can be recovered by returning to a high-fiber diet.
Non-antibiotic xenobiotics	[69–73]	Drugs including actominopin, proton pump inhibitors, and metformin alter the microbiome. Microbial metabolism may contribute to side effects associated with drugs.
Genetics	[3, 66, 67]	Identical twins have microbiomes that have more similarity than those of fraternal twins. Some clades are heritable, although the heritability varies. Microbes that coevolved with an ancestral group may be better symbionts.
Exercise	[63–65]	Extreme athletes have different microbiomes than sex-, age-, and weight-matched controls. It is, however, difficult to separate the effect of diet from the effect of exercise. Mouse models suggest that exercise alone has an impact.
Pet ownership and cohabitation	[68]	Individuals living together—whether genetically related or unrelated—share more of their microbiomes than people who do not cohabitate. Pets act as vectors, although their largest effect is on the skin microbiome.
Small		
Short-term dietary intervention	[61, 74]	Short-term diet may change microbial communities, but they return to the previous configuration once the intervention has ended.

Many comparative studies of mammals have shown that composition of the gut microbial community varies strongly with diet, a trait that tends to be conserved within animal taxonomic groups [38–40]. For example, in a landmark study of the gut microbiomes of major mammalian groups, Ley et al. [41] showed that diet classification explained more variation across diverse mammalian microbiomes than any other variable (although different gut physiologies are generally adapted to different diets, so separating these variables is difficult). However, a separate study of foregut and hindgut fermenting avian and ruminant species found that gut physiology explained the largest amount of gut microbiome variation [42], suggesting that diet may have been a confounding variable. More studies are now beginning to tease apart the relative effects of diet and other factors, such as taxonomy, by considering multiple animal lineages, such as panda bears and baleen whales, that have diets that diverge from those of their ancestors [43, 44].

Even within a single species, diet has been shown to shape the gut microbial community significantly. In humans, for example, changes in the gut microbiome associated with diet shifts in early development are consistent across populations, as the microbiomes of infants and toddlers systematically differ from those of adults [45, 46]. Although the microbiome continues to change over the course of a person’s life, the magnitudes of differences over time are much smaller in adults than in infants. The early differences are, in part, due to changes in diet, although it may be hard to decouple diet-specific changes from overall developmental changes. The microbiome developmental trajectory for infants may begin even before birth: the maternal gut and vaginal microbiome change during pregnancy. The gut microbiome of mothers in the third trimester, regardless of health status and diet, enters a proinflammatory configuration [47]. The vaginal microbiome has reduced diversity and a characteristic taxonomic composition during pregnancy [48, 49], which may be associated with the transfer of specific beneficial microbes to the infant. During delivery, neonates acquire microbial communities that reflect their delivery method. The undifferentiated microbial communities of vaginally delivered babies are rich in *Lactobacillus*, a common vaginal microbe, whereas those of infants born by cesarean are dominated by common skin microbes including *Streptococcus* [50].

Over the first few months of life, the infant microbiome undergoes rapid changes [46], some of which correlate with changes in breast milk composition and the breast milk microbiome [51]. Formula-fed infants also have microbial communities that are distinct from those of breastfed babies [52, 53]; formula was associated with fewer probiotic bacteria and with microbial communities closer than those of breastfed babies to the microbial communities of adults. The introduction of solid food has been associated with dramatic changes in the microbiome, during which toddlers come to more closely resemble their parents [45, 46, 52]. The compositional difference between infants and adults is larger than the differences resulting from compounded technical effects across studies [34], suggesting that this difference between human infants and adults is one of the largest effects on gut microbial community in humans.

Within children and adults, studies suggest that changes in the gut microbiome could stem from dietary changes corresponding to technological advancement, including shifts from a hunter-gatherer to an agrarian or industrialized society [45, 54]. These differences may be confounded, however, by other non-diet-related factors that co-vary with these shifts, such as exposure to antibiotics [55, 56] or the movement of industrialized individuals into confined, more sterile buildings [57]. Antibiotic-induced changes in the microbiome can last long after the course of treatment is completed [56, 58]. Although differences in microbial communities resulting from antibiotic use can be seen [56], different individuals respond differently to a single antibiotic [59]. At this scale, some technical effects, such as those associated with differences in sequencing platforms or reagent contamination, are smaller than the biological effect and can be corrected for using sequence data processing and statistical techniques. Nevertheless, compounded effects may lead to differences between studies that are larger than the biological effect being examined. It is often possible to see clear separation between communities using Principal Coordinates Analysis (PCoA) space even with cross-sectional data. PCoA provides a quick visualization technique for assessing which effects are large and which are small in terms of the degree of difference in a reduced-dimensionality space, although statistical confirmation using techniques such as ANOSIM or PERMANOVA is also necessary. Essentially, factors that led to groups of samples separating more in PCoA space have larger effects. One important caveat is that the choice of distance metric can have a large effect on this clustering [60].

On a finer scale, for example when considering only Western human populations, the effects of individual diet are less pronounced. Long-term dietary patterns, however, have been shown to alter the microbiome [61]. Several mouse models have demonstrated a mechanistic role for diet. In one study, mice were humanized with stool from lean or obese donors. Cohousing obese mice with lean mice led to weight loss only if the obese mouse was fed a high-fiber diet [2]. Another study using humanized gnotobiotic mice (that is, initially germ-free mice colonized with human-derived microbes) showed that a low-fiber diet led to a significant loss of diversity, and that the changes in the microbiome were transmitted to pups [62]. Increasing the fiber in the mouse’s diet led to an increase in microbiome diversity [62]. Nevertheless, it can be hard to separate long-term dietary patterns from other factors that shape individual microbial communities. For example, exercise is hypothesized to alter the microbiome [63–65]. One study found differences between extreme athletes and age- and weight-matched controls [64]. It is unclear, however, whether these differences are due to the strenuous training regime, the dietary requirements of the exercise program, or a combination of these two factors [63, 64]. At this scale, cross-sectional data may overlap in PCoA space.

Host genetics help to shape microbial communities. Identical twins share slightly more of their overall microbial communities than do fraternal twins [3, 66], although some taxa are far more heritable than others. Cross-sectional studies suggest that the coevolution of bacteria and human ancestors can also shape disease risk: the transfer of *Helicobacter pylori* strains that evolved separately from their host may confer a higher risk of gastric cancer [67]. However, separating the effect of genetics from those of vertical transmission from mother to child [52] or of transfer due to cohabitation with older children can be difficult, and the relative effect sizes of these factors is unknown [68].

Cohabitation and pet ownership modify microbial communities, and their effects can be confounded with those of diet (which is often shared within a household). Spouses are sometimes used as controls, because they are hypothesized to have similar diets. However, cohabitating couples can share more of their skin microbiomes, and to a lesser extent their gut microbiomes, than couples who do not live together [68]. Dog ownership also influences the similarity of the skin, but not fecal, microbial community [68].

Exposure to chemicals other than antibiotics also shapes our microbiome, and microbes may in turn shape our responses to these chemicals. There is mounting evidence that use of pharmaceuticals—both over-the-counter [69] and prescription [70–73]—leads to changes in microbial community structures. For example, metformin use was correlated with a change in the microbiome of Swedish and Chinese adults with type II diabetes [72]. (Notably, in this study, the failure to reproduce taxonomic biomarkers that were associated with disease in the two populations was due to different prevalence of metformin use, which has a large effect on the microbiome; the drug was used only in diabetes cases and not in healthy controls.) Changes in the microbiome may also be linked to specific side effects; for example, metformin use improved not only glucose metabolism but also pathways contributing to gas and intestinal discomfort. Which of these factors contributed most to microbiome changes is difficult to resolve with the available data [72].

Within a single individual, short-term or long-term interventions present the largest potential for remediation, but the effects of interventions often vary and methodology matters. A study that looked for a consistent change in the microbiome in response to a high- or low-fiber diet found no differences [43]. A group focusing on a mostly meat or mostly plant diet found a difference in community structure only when considering relative change in community structure, and did not find that communities from different people converged on a common state overall [74].

## Technical factors affecting the microbiome

**Fig. 1:**
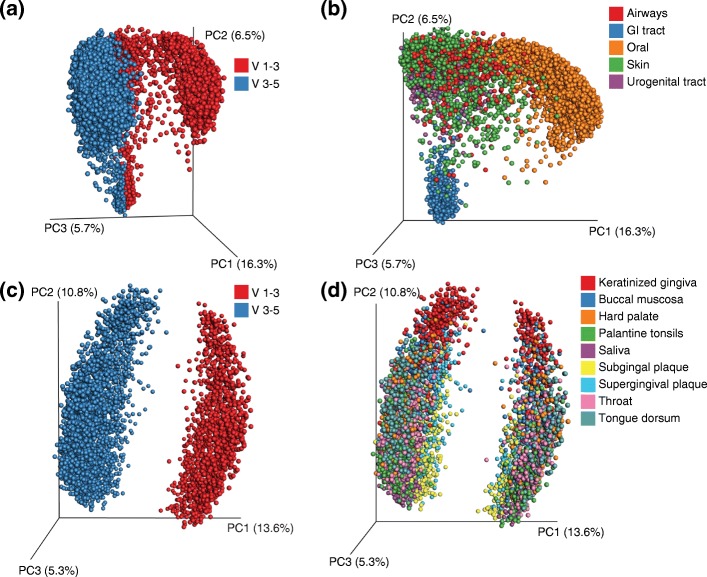
PCoA differences in PCR primers can outweigh differences among individuals within one body site, but not the differences between different body sites. In the Human Microbiome Project (HMP) dataset, when V1-3 and V3-5 primers are combined across body sites, **a** the effect of PCR primers is small compared to **b** the effect of body site. However, if we analyze individual body sites such as **c** the mouth or **d** the mouth subsites, the effect of primer is much greater than the difference between different individuals (or even of different locations within the mouth) at that specific body site. *GI* gastrointestinal

Technical sources of variation have a large influence on the observed structure of the microbial community, often on scales similar to or larger than biological effects. Considerations include sample collection and storage techniques, DNA extraction method, selection of hypervariable region and PCR primers, sequencing method, and bioinformatics analysis method (Fig. 1, Table 2).

**Table 2:** Technical factors affecting the microbiome

Covariate	References	Findings
Sample storage	[76–79]	The gold standard for storage is −80 °C. Long-term storage at room temperature or multiple freeze-thaw cycles alter community stability. Room temperature preservation methods improve stability but may alter microbial community structure.
Primers and sequencing method	[32, 34, 82, 83]	Primer selection and hypervariable region influence the observed microbial community. Resolution is better with longer reads and the V2 and V4 regions of the 16S rRNA.
Extraction kit and kit lot	[80, 81, 90, 91]	Extraction kit alters the observed community by increasing the probability that certain bacteria will be observed. In low-biomass samples, reagent contamination in the extraction kit can have a larger effect on the observed community than the biological effect of interest.
Bioinformatics	[22, 61, 74, 84, 85, 88]	Clustering method, choice of reference, chimera removal, or de-noising method and quality filtering influence results and taxonomic assignments. Additionally, the choice of statistical analysis and data visualization can lead to conflicting conclusions with similar data.

An early consideration in microbiome studies is sample collection and storage. Stool samples can be collected using a bulk fecal sample or a swab from used toilet paper [75]. The gold standard for microbial storage is freezing samples at −80 °C. Recent studies suggest that long-term storage at room temperature can alter sample stability. Preservation methods such as fecal occult blood test cards, which are used in colon cancer testing [76, 77], or storage with preservatives [76] offer better alternatives. Freeze-thaw cycles should be avoided because they affect reproducibility [78]. Nevertheless, some studies have found that preservation buffers alter the observed community structure [79]. Preservation method seems to have a larger impact on observed microbial communities than collection method, although it is not sufficient to overcome inter-individual variation [76].

**Fig. 2:**
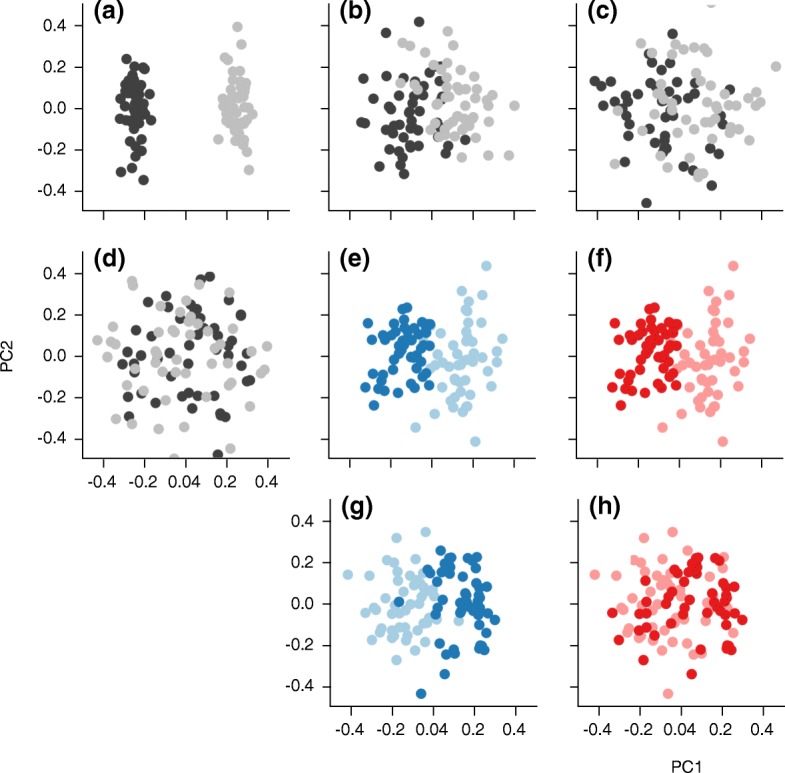
PCoA patterns of technical and biological variation. Two groups (*black*, *gray*) with significantly different distances (*P* < 0.05) and varying effect size. **a** A large separation in PCoA space and large effect size. Separation in PCoA space (shown here in the first two dimensions) may be caused by technical differences in the same sample set, such as different primer regions or sequence lengths. **b** Clear separation in PCoA space, similar to patterns seen with large biological effects. In cross-sectional studies, age comparisons between young children and adults or comparisons between Western and nonWestern adults might follow this pattern. **c** Moderate biological effect. **d** Small biological effect. Sometimes effects can be confounded. In **e** the technical effect and in **f** the biological effect are conflated because the samples were not randomized. In **g** and **h**, there is a technical and a biological effect, but the samples were randomized among conditions, so the relative size of these effects can be measured

Sample processing plays a large role in determining the observed microbiota. DNA extraction methods vary in their yields, biases, and reproducibility [80, 81]. For example, the extraction protocols used in the Human Microbiome Project (HMP) and the European MetaHIT consortium differed in the kingdoms and phyla extracted [81]. Similarly, the DNA target fragment and primer selection can create biases. Although the V2 and V4 regions of the 16S rRNA gene are better than others for broad phylogenetic classification [82], these regions often yield results that differ from each other, even when combined with mapping to a common set of full-length reference sequences. For example, all the HMP samples were sequenced using primers targeting two different hypervariable regions of the 16S rRNA gene [83]. The separation of samples in PCoA space indicates that the technical effect of different primer regions is larger than any of the biological effects within the study (Fig. 2). Finally, the choice of sequencing technology also has an effect on the observed community structure. Longer reads can improve classification accuracy [82], but only if the sequencing technology does not introduce additional errors.

Choices in data processing also play a role in the biological conclusions reached in a study or set of combined studies. Read trimming may be necessary to normalize combined studies [34], but shorter reads can affect the accuracy of taxonomic classifications [82]. The selection of a method to map sequences into microbes has a large impact on the microbial communities identified. Several approaches exist, but clustering of sequences into Operational Taxonomic Units (OTUs) on the basis of some threshold is common. Sequences may be clustered against themselves [22, 84], clustered against a reference [84], or clustered against a combination of the two [85]. The selection of a particular OTU clustering method and OTU clustering algorithm alters the observed microbial community and can artificially inflate the number of OTUs observed [22, 84]. De-noising (a technique commonly used with 454 sequencing [22]), removal of chimeric sequences generated during PCR [86, 87], and quality filtering of Illumina data can help to alleviate some of these problems [24, 88]. After OTU picking, the selection of biological criteria, ecological metric, and statistical test can lead to different biological conclusions [60, 89].

The degree to which technical variation impacts biological conclusions depends on the relative scale of the effects and the method of comparison. For very large effects, biologically relevant patterns may be reproducible when studies are combined even though there is technical variability. A comparison of fecal and oral communities in adult humans may be robust to multiple technical effects, such as differences in extraction method, PCR primers, and sequencing technology (Fig. 2). Conversely, subtle biological effects can quickly become swamped. Many biological effects of interest to current research have a smaller effect on observed microbial communities than the technical variations commonly observed among studies [32, 34].

Failure to consider technical variation can also confound biological interpretation. In low-biomass samples, technical confounders such as reagent contamination can have larger effects than the biological signal. A longitudinal study of nasopharyngeal samples from young children [90] exemplified this effect. Principal Coordinates Analysis of the data found a sharp distinction by age. It was later determined, however, that the samples had been extracted with reagents from two different lots—the differences in the microbial communities were due to reagent contamination and not biological differences [91]. Higher biomass samples are not immune to this problem. Extraction of case and control samples using two different protocols could potentially lead to similar erroneous conclusions.

## Comparing effects: the importance of large integrated studies

**Fig. 3:**
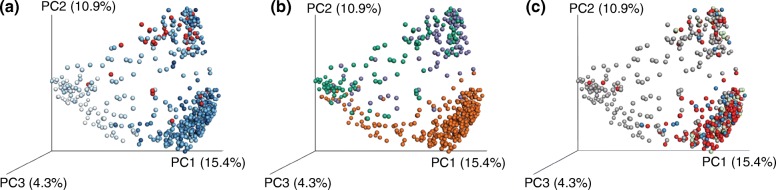
Relative effect sizes of biological covariates on the human microbiome. Principal coordinates projection of unweighted UniFrac distance, using data from Yatsunenko et al. [45], shows **a** age (*blue* gradient; missing samples in *red*) separating the data along the first axis and **b** country (USA, *orange*; Malawi, *green*; Venezuela, *purple*) separating the data along the second principal coordinates axis. **c** Body mass index in adults has a much more subtle effect, and does not separate along any of the first three principal coordinate axes (normal, *red*; overweight, *green*; obese, *blue*; missing samples, *gray*)

Large-scale integration provides a common framework for comparing effects. Studies of large populations are often successful in capturing the significance of biological patterns such as age [45], human microbiome composition [75, 92], or specific health conditions such as Crohn’s disease [93]. The scale of the population means that multiple effects can also be compared across the same set of samples. For example, the HMP provided a reference map of microbial diversity found in the body of Western adults [92]. Yatsunenko et al. [45] highlight the effect of age over other factors including weight and country of origin, demonstrating that age has a larger effect on the microbiome than nationality, which in turn has a larger effect than weight (Fig. 3). Two recently published studies of Belgian and Dutch populations provide very interesting examples of what can be achieved through larger population-based studies, especially in terms of understanding which factors are important in structuring the microbiome.

The LL-Deep study, which used both 16S rRNA amplicon sequencing and shotgun metagenomic sequencing on a cohort of 1135 Dutch individuals, associated 110 host factors to 125 microbial species identified by shotgun metagenomics. In particular, this study found that age, stool frequency, dietary variables such as total carbohydrates, plants and fruits, and fizzy drinks (both 'diet' brands and those with sugar) had large effects, as did drugs such as proton pump inhibitors, statins, and antibiotics [94]. Interestingly, the authors observed 90 % concordance in associations between the shotgun metagenomic and the rRNA amplicon results, suggesting that many conclusions about important microbiome effects may be robust to some kinds of methodological variation, even if the absolute level of specific taxa are not. The Flemish Gut Flora Project, which used 16S rRNA amplicon sequencing on a cohort of 1106 individuals, identified 69 variables relating to the subjects that correlated with the microbiome, including use of 13 drugs ranging from antibiotics to antidepressants, and explained 7.7 % of the variation in the microbiome. The consistency of the stool (which is a proxy for transit time), age, and body mass index were especially influential, as was the frequency of fruit in the diet; the adult subjects did not show effects of early-life variables such as delivery mode or residence type during early childhood [95]. The American Gut Project (www.americangut.org), now with over 10,000 samples processed, is a crowd-sourced microbiome study that expands on the effects considered by the HMP to evaluate microbial diversity across Western populations with fewer restrictions on health and lifestyle. Large-scale studies have two advantages for comparisons. They can help to limit technical variability because samples within the same study are collected and processed in the same way. This reduces technical confounders, making it easier to draw biological conclusions. Second, large population studies increase the probability of finding subtle biological effects which may be lost in the noise of smaller studies.

Meta-analyses that place smaller studies into the context of these larger studies can also provide new insights into the relative size of the changes seen in the smaller studies [34]. Weingarden et al. [96] took advantage of the HMP and contextualized the dynamics of fecal material transplants (FMT). Their initial data set focused on a time series from four patients who had recurrent *Clostridium difficile* infection and a healthy donor. By combining the time series results with a larger dataset, they revealed the dramatic restoration that diseased patients undergo after the transplant is administered, ultimately helping the patients recover from the severe *C. difficile* infection [96, 97].

When conducting a meta-analysis, however, it is important to consider whether the differences in microbial communities in different studies are due to technical or biological effects. Selecting studies that each include biologically relevant controls can help to determine whether the scale of the effect between the studies results from a biological or a technical covariate. In the FMT study [96], the donor (control) sample clustered with the HMP fecal samples, while the pre-treatment recipients did not. Had the donor point grouped somewhere else, perhaps among the skin samples or in a completely separate location, it could have indicated a large technical effect, suggesting that the studies should not be combined into a single PCoA (although trends might still be identified within each study and compared). Similarly, a study of the progression of the microbiome of an infant during the first 2 years of life showed changes in the infant microbiome with age [36], but it was only when this study was placed in the context of the HMP that the scale of developmental change within a single infant body site relative to differences in the microbiome among distinct human body sites became clear [34].

## Leveraging effect size in meta-analysis

Compared to other fields, meta-analysis among microbiome studies is still in its infancy. Statistical methods can help to overcome the complication of technical effects in direct comparisons, allowing focus on the biological results. Medical drug trials [98, 99] routinely report quantified effect sizes. This practice has several advantages. First, it moves away from a common binary paradigm of not significant or significant at *P* < 0.05 [35]. The combination of significance and effect size can be important for avoiding undue alarm, as has been shown in other fields. For instance, a recent meta-analysis found a statistically significant increase in cancer risk associated with red meat consumption [100]. The relative risk of colon cancer associated with meat consumption is, however, much lower than the relative risk of colon cancer associated with an inflammatory bowel disease (IBD) diagnosis. With a *P* value alone, it might not have been possible to determine which factor had a larger impact on cancer risk. Effect size quantification may also help to capture the range of variation in effects across different populations: there are probably multiple ways for a microbial community to be 'sick', rather than single set of taxa that are enriched or depleted in perturbed populations. We see this, for example, in the different 'obese' microbiomes that seem to characterize different populations of obese individuals. Finally, effect size is also closely linked to statistical power, or the number of samples needed to reveal a statistical difference. Quantitative power estimates could improve experimental design and limit publication bias [35].

Unfortunately, effect size and statistical power are challenging to calculate in microbiome data. Currently, applied power calculations (reviewed in [35]) typically make assumptions about the data that do not hold true in the analysis of microbial communities (Box 1). Some solutions to this problem have been proposed, including the Dirichlet Multinomial method [101] and random forest analysis [102] for OTUs, a simulation-based method for PERMANOVA-based beta diversity comparisons [103], and power estimation by subsampling (Box 1). Nevertheless, power analysis remains rare in microbiome studies. New methods could facilitate better understanding of effect sizes. As the scope of microbiome research continues to expand to include metabolomic, metagenomics, and metatranscriptomic data, effect size considerations will only become more important.

## Considerations for study design

Large-scale studies provide insight into which variables have broad effects on the microbiome, but they are not always feasible. Small, well-designed studies that address hypotheses of limited scope have a large potential to advance the field. In designing one of these studies, it is better to define a population of interest narrowly, rather than trying to draw general conclusions. The design and implementation of small studies should strive for four goals: limited focus, rich metadata collection, appropriate sample size, and minimized technical variation.

Limiting the scope of the study increases the probability that a small study will be successful because it decreases noise and confounding factors. For example, the hypothesis 'milk consumption alters the microbial community structure and richness in children' might be better phrased as 'milk consumption affects the microbial community structure and richness in children in third through fifth grade attending New York Public schools'. Additionally, the study should define exclusion criteria; for example, perhaps children who have taken antibiotics in the past 6 months or 1 year should be excluded [56, 58]. Broader hypotheses may be better tackled in meta-analyses, where multiple small, well-designed studies on a similar topic can be combined.

Information about factors that might influence the microbiome should be included in sample collection. For example, the study of children attending New York City Public Schools might not have birth delivery method as an exclusion criterion, but whether the child was born by C-section or vaginally could influence their microbial community, so this information should be recorded and analyzed. Self-reported data should be obtained using a controlled vocabulary and common units. If multiple small studies are planned, standard metadata collection will minimize time in meta-analysis.

A second consideration in defining scope is to identify a target sample size. Other studies may be used as a guide, particularly if the data can be used to quantify an effect size. Quantitative power calculations (Box 1) can be particularly helpful in defining a sample size. Nevertheless, this comparison should be done judiciously. Sample sizes should be estimated by selecting a known effect that is expected to be of similar scale. It may be prudent to consider the phenotype associated with the effect, and whether the effect might directly target microbes. For example, one might guess that a new drug that inhibits folate metabolism, which is involved in DNA repair in bacteria and eukaryotes, might have an effect close to those of other drugs that are genotoxic, such as specific classes of antibiotics and anticancer agents.

Technical variation within a study should be minimized. Sample collection and storage should be standardized. Studies in which samples cannot be frozen within a day of collection should consider a preservation method, although even preserved samples should be frozen at −80 °C for long-term storage [76, 77]. If possible, samples should be processed together using the same reagents. If this is not possible because of the size of the study, samples should be randomized to minimize the confounding of technical and biological variables [91]. The use of standard processing pipelines, like those described by the Earth Microbiome Project [104, 105], may facilitate data aggregation for meta-analyses. Participation in standardization efforts, such as the Microbiome Quality Control Project (http://www.mbqc.org/) and the Unified Microbiome Initiative [106], can help to identify sources of lab-to-lab variation.

## Conclusions

Microbiome research is rapidly advancing, although several challenges that have been tackled in other fields, including epidemiology, ecology, and human genetic studies (in particular, genome-wide association studies), need to be addressed fully. First, technical variation still makes it difficult to compare claimed effect sizes, or claimed associations of particular taxa with particular phenotypes. Standardized methods, including bioinformatics protocols, will help immensely here. This is particularly an issue for translational studies between humans and animal models, because it can be difficult to determine whether differences in microbial communities or host responses to these changes are due to differences in the host physiology or variation in the variable of interest. However, the potential payoff for translation of microbiome results from high-throughput animal models, such as flies or zebrafish, to humans, is enormous.

In this review, we have focused mainly on 16S rRNA amplicon analysis and shotgun metagenomic studies because these are most prevalent in the literature at present. However, microbiome studies are continuing to expand, such that a single study can include multi-omics techniques such as metatranscriptomics, metaproteomics, and metabolomics. Before we embark too far on the exploration of multiomics datasets, methods standardization across multiple platforms will be necessary to facilitate robust biological conclusions, despite the considerable cost of such standardization efforts.

Overall, the field is converging on many conclusions about what does and does not matter in the microbiome: improved standards and methodologies will greatly accelerate our ability to integrate and trust new discoveries.

## Box 1. Methods for power analysis of microbiome data

The calculation of effect size in microbiome data is challenging for several reasons. Operational Taxonomic Unit (OTU)-based methods are affected by the sparsity of OTUs, meaning that many samples may not contain a given taxon. This means that OTUs do not fit the Gaussian distribution and/or non-correlated observation assumptions required for common statistical tests, such as *t* tests. While many methods exist to evaluate differences in OTUs (reviewed in [107]), currently only one defines power-based calculations.

The Dirichlet Multinomial method [101] models the variability and frequency of an OTU within a population or across populations. The data are fitted to a modified multinomial distribution. La Rosa et al. [101] developed power and effect size calculations for the Dirichlet multinomial model based on Cramer’s model for the chi-square distributions [108]. A second technique for OTU-based comparison is the application of random forest models for supervised regression and classification. Random forest excels at feature selection, identifying the most relevant OTUs that are correlated with metadata and ranking features with their contribution to the model. Power can be estimated by a learning curve, comparing how well these features predict the metadata category against the number of samples used in the training set.

Effect size calculations for diversity metrics, particularly beta diversity, are also challenging because permutative tests are required. For common parametric tests, power is defined on the basis of the distribution of the test statistic [109]. Nonparametric tests, including permutative tests, do not have a defined distribution for the test statistic, so power is difficult to calculate [110, 111].

An emerging solution to effect size estimation is the use of simulation to estimate statistical power. Kelly et al. [103] proposed that power could be calculated from PERMANOVA tests by estimating an effect size on the basis of the original data, using an ANOVA-based estimator. They then simulated distance matrices with the same properties as the original dataset, and estimated power by bootstrapping the simulated distance matrices.

A second solution involves subsampling the data. The Evident software package (https://github.com/biocore/Evident) relies on subsampling the data to estimate visual separation between groups. Monte Carlo simulations are used to estimate the variance in a data cloud, and provide an estimate of visual separation. The package allows exploration of both the sampling depth and the number of samples. An extension of the Evident protocol is to apply the same subsampling procedure to a statistical test as an estimate of power. This solution has been implemented in the scikit-bio software package (http://scikit-bio.org/).

## Abbreviations

FMT: Fecal material transplants
HMP: Human microbiome project
OTU: Operational taxonomic unit
PCoA: Principal coordinates analysis
